# Albumin orchestrates a natural host defense mechanism against mucormycosis

**DOI:** 10.21203/rs.3.rs-5441197/v1

**Published:** 2024-12-03

**Authors:** Antonis Pikoulas, Ioannis Morianos, Vassilis Nidris Nidris, Rania Hamdy, Angeles López-López, Maria Moran-Garrido, Valliappan Muthu, Maria Halabalaki, Maria Papadovasilaki, Kyrmizi Irene, Yiyou Gu, Robina Aerts, Toine Mercier, Yuri Vanbiervliet, Sung-Yeon Cho, Amy Spallone, Dimitrios Samonakis, Efstathios Kastritis, Elias Drakos, Maria Tzardi, Aristides Eliopoulos, Konstantina Georgila, Agostinho Carvalho, Oliver Kurzai, Shivaprakash Rudramurthy, Fanny Lanternier, Kyriacos Petratos, Johan Maertens, Vincent Bruno, Dimitrios Kontoyiannis, Coral Barbas, Sameh Soliman, Ashraf Ibrahim, Georgios Chamilos

**Affiliations:** School of Medicine, University of Crete; IMBB, FORTH, Nikolaou Plastira 100 GR-70013, Heraklion, Crete GREECE; School of Medicine, University of Crete; Research Institute for Medical and Health Sciences, University of Sharjah, Sharjah, United Arab Emirates; Centro de Metabolómica y Bioanálisis (CEMBIO), Facultad de Farmacia, Universidad San Pablo-CEU, CEU Universities, Urbanización Montepríncipe, 28660 Boadilla del Monte, Ma; Centro de Metabolómica y Bioanálisis (CEMBIO), Facultad de Farmacia, Universidad San Pablo-CEU, CEU Universities, Urbanización Montepríncipe, 28660 Boadilla del Monte, Ma; Department of Pulmonary Medicine, Postgraduate Institute of Medical Education and Research (PGIMER), Chandigarh, India; Department of Pharmacy, National and Kapodistrian University of Athens, Athens, Greece; IMBB, FORTH, Nikolaou Plastira 100 GR-70013, Heraklion, Crete GREECE; Department of Medicine, University of Crete; The Lundquist Institute for Biomedical Innovation, Harbor-UCLA Medical Center; Department of Hematology, University Hospitals Leuven, Leuven, Belgium; Department of Hematology, University Hospitals Leuven, Leuven, Belgium; Department of Hematology, University Hospitals Leuven, Leuven, Belgium; Department of Infectious Diseases, Infection Control and Employee Health, University of Texas MD Anderson Cancer Center, Houston, TX 77030, USA; Department of Infectious Diseases, Infection Control and Employee Health, University of Texas MD Anderson Cancer Center, Houston, TX 77030, USA; School of Medicine, University of Crete; School of Medicine, National and Kapodistrian University of Athens, Athens, Greece; School of Medicine, University of Crete; School of Medicine, University of Crete and University Hospital; School of Medicine, National and Kapodistrian University of Athens, Athens, Greece; School of Medicine, National and Kapodistrian University of Athens, Athens, Greece; University of Minho; Institute for Hygiene and Microbiology, University of Würzburg, 97080, Würzburg, Germany; Department of Medical Microbiology, Postgraduate Institute of Medical Education and Research (PGIMER), Chandigarh, India; Service de Maladies Infectieuses et Tropicales, Hôpital Universitaire Necker-Enfants Malades, Assistance Publique-Hôpitaux de Paris, Université Paris Cite, France; Forth Institute of Molcular Biology and Biotechnology; Department of Hematology, University Hospitals Leuven, Leuven, Belgium; University of Maryland School of Medicine; The University of Texas MD Anderson Cancer Center; Centro de Metabolómica y Bioanálisis (CEMBIO), Universidad San Pablo-CEU; University of Sharjah; The Lundquist Institute at Harbor-University of California Los Angeles Medical Center; Institute of Molecular Biology and Biotechnology, FORTH, Hellas

**Keywords:** Albumin, Free fatty acids, Mucorales, Rhizopus, mucormycosis, mucoricin, fungi, serum, host defense

## Abstract

Mucormycosis is an emerging, life-threatening human infection caused by fungi of the order Mucorales. Metabolic disorders uniquely predispose an ever-expanding group of patients to mucormycosis via poorly understood mechanisms. Therefore, it is highly likely that uncharacterized host metabolic effectors confer protective immunity against mucormycosis. Here, we uncover a master regulatory role of albumin in host defense against Mucorales through the modulation of the fungal pathogenicity program. Our initial studies identified severe hypoalbuminemia as a prominent metabolic abnormality and a biomarker of poor outcome in independent cohorts of mucormycosis patients. Strikingly, we found that purified albumin selectively inhibits Mucorales growth among a range of human pathogens, and albumin-deficient mice display susceptibility specifically to mucormycosis. The antifungal activity of albumin is mediated by the release of bound free fatty acids (FFAs). Importantly, albumin prevents FFA oxidation, which results in loss of their antifungal properties. A high degree of FFA oxidation is found in the sera of patients with mucormycosis. Physiologically, albumin-bound FFAs blocks the expression of the mycotoxin mucoricin and renders Mucorales avirulent in vivo. Overall, we discovered a novel host defense mechanism that directs the pathogen to suppress its growth and the expression of virulence factors in response to unfavorable metabolic cues regulated by albumin. These findings have major implications for the pathogenesis and management of mucormycosis.

Mucorales fungi cause mucormycosis: an emerging, life-threatening, opportunistic infection with limited therapeutic options and incompletely understood pathogenesis^1,6,7^. The overall mortality of mucormycosis exceeds 50% and approaches 100% in patients with disseminated disease^1^. The clinical hallmark of mucormycosis is massive tissue necrosis induced by the fungus, which impedes antifungals to reach the sites of infection and often necessitates radical, disfiguring surgery to control the disease^1,8^. In contrast to other fungal infections, mucormycosis predominantly affects an ever-expanding group of patients with metabolic abnormalities through incompletely characterized mechanisms^1,4,5^. Specifically, poorly controlled diabetes mellitus (DM), acidosis, acquired iron overload syndromes, malnutrition, and immunometabolic dysregulation induced by COVID-19, uniquely predispose individuals to the development of mucormycosis^1–4,6,9^. Therefore, uncharacterized metabolic host defense mechanisms may confer protective immunity against mucormycosis.

From the pathogen perspective, Mucorales senses cues in the tissue environment, which triggers the expression of virulence factors that transform these saprophytic organisms into rapidly invasive and potentially lethal pathogens^1^. Specifically, the production of the potent mycotoxin mucoricin during Mucorales hyphae growth^10^ induces extensive tissue necrosis^11^, whereas the binding of CotH invasins to specific host receptors promotes angioinvasion and fungal dissemination^12–14^. Interestingly, germinating spores of Mucorales evade phagocytosis and induce acute lethality within 24 hours of pulmonary infection in immunocompetent mice^15^. Thus, it is essential to identify metabolic host effectors that prevent extracellular growth and modulate Mucorales pathogenicity during the early stages of infection.

In this study, we discovered a master regulatory role of albumin in anti-Mucorales host defense. Specifically, we found that albumin exhibits potent and distinct *in vitro* inhibitory activity against Mucorales, which is mediated by bound free fatty acids (FFAs). Mechanistically, we show that albumin-bound FFAs inhibit the expression of mucoricin during Mucorales growth, rendering the fungus avirulent *in vivo,* and that albumin deficient mice display selective susceptibility to mucormycosis and impaired antifungal activity of their sera. Finally, we identify albumin level as surrogate of the inhibitory activity of serum against Mucorales and a promising biomarker of mucormycosis outcome. Collectively, our data suggest a new model of metabolic host defense that prevents Mucorales infection by modulating fungal pathogenicity and is regulated by albumin, with profound implications for pathogenesis and treatment of mucormycosis.

## Selective inhibitory activity of human albumin against Mucorales and association of serum albumin level with mucormycosis outcome.

Since the early 1960s, human serum has been shown to have important inhibitory effects against Mucorales, which remain molecularly unexplored^16,17^. We found that, when compared to healthy individuals, the ability of sera from patients with mucormycosis to inhibit hyphal growth of a clinical isolate of *Rhizopus arrhizus var. R. delemar* (hereafter *R. delemar*) is almost completely lost **(Extended Data Fig. 1)**. Albumin, the most abundant serum protein, regulates important physiological functions intravascularly, in the interstitial space, and on mucosal surfaces^18^. Furthermore, severe hypoalbuminemia is a common finding in patients with diverse immunometabolic abnormalities predisposing for mucormycosis^2,5,19^. Therefore, we decided to comprehensively evaluate the physiological function of albumin against Mucorales.

**Figure 1:**
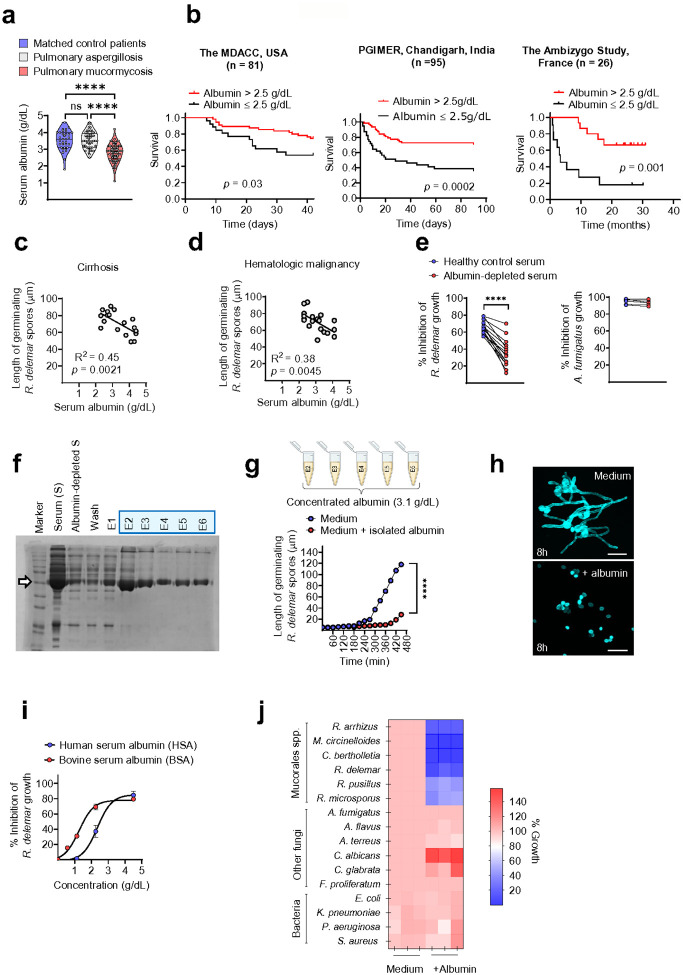
Specialized activity of albumin against Mucorales. **(a)** Serum albumin levels on the time of diagnosis in contemporaneously matched controls (n = 33), patients with pulmonary aspergillosis (n = 50) and patients with mucormycosis (n = 97). ****p < 0.0001, one-way ANOVA and Tukey’s multiple comparisons post hoc test. **(b)** Kaplan–Meier survival curves of mucormycosis patients with severe hypoalbuminemia (≤ 2.5 g/dL) compared with those of other mucormycosis patients from clinical cohorts in the USA (n = 81), India (n = 95) and France (n = 26). The nonparametric log-rank (Mantel–Cox) test was used to calculate differences between survival curves. **(c, d)**Correlation between serum albumin levels and *R. delemar* growth in human sera obtained from patients with **(c)** cirrhotic disease (n = 18) or **(d)**hematologic malignancies (n = 20). **(e)** Assessment of the inhibitory effects of albumin depletion from human serum obtained from healthy individuals on *R. delemar*(n = 15) and *A. fumigatus* growth (n = 6). ****p < 0.0001, Wilcoxon matched-pairs signed rank test. **(f)** Human serum and albumin-rich eluted fragments were analysed via Coomassie blue staining. **(g)** Length of germinating *R. delemar* spores cultured in medium or medium supplemented with albumin isolated from human serum, as assessed by time lapse microscopy ****p < 0.0001, multiple unpaired t tests**. (h)**Representative fluorescence images of Calcofluor White-labelled *R. delemar* spores cultured for 8 h in regular media or media supplemented with albumin. Scale bar, 50 mm. **(i)**Inhibition of *R. delemar* growth by increasing concentrations of human serum albumin (HSA) or bovine serum albumin (BSA). **(j)** Quantification of the growth of different Mucorales species and other bacterial and fungal human pathogens cultured in regular media or media supplemented with albumin.

We initially explored the associations of serum albumin levels with susceptibility to the development of pulmonary mucormycosis and its outcome in contemporaneous high-risk hematological malignancy patients at a major tertiary care cancer center in the US (**Supplementary Table 1**). Interestingly, most patients who developed pulmonary mucormycosis had hypoalbuminemia (< 3.5 g/dL) and significantly lower albumin levels at diagnosis when compared to control patients matched for the underlying disease who developed bacterial pneumonia or pneumonia caused by the major airborne human fungal pathogen *Aspergillus fumigatus* (Fig. 1a). Furthermore, patients with mucormycosis and very low albumin levels (≤ 2.5 g/dL) had significantly lower survival rates when compared to other mucormycosis patients (Fig. 1b). These findings were independently validated through an analysis of serum albumin levels in a cohort of patients with pulmonary mucormycosis in a tertiary care center from PGIMER, Chandigarh, India (Fig. 1b and **Supplementary Table 1**), who had DM and COVID-19 as main underlying risk factors, and a published cohort of patients with mucormycosis from France (Ambizygo Study^20^) who had different risk factors (Fig. 1b).

We then analyzed the functional relationship between the serum albumin concentration and antifungal activity in patients at high risk for mucormycosis. Interestingly, we detected a significant association between the degree of hypoalbuminemia and the loss of inhibitory activity of serum against *R. delemar* hyphal formation in prospectively collected sera from patients with liver cirrhosis (Fig. 1c and **Supplementary Table 1**) or hematological malignancy (Fig. 1d and **Supplementary Table 1**).

To account for potential confounders related to the underlying disease, we performed albumin depletion in sera from healthy individuals via affinity chromatography^21^. Equilibration of Cibacron blue chromatography columns was achieved with concentrated serum filtrate previously passed through a 45 kDa centrifugal filter unit, to ensure that the albumin-depleted flow through was not diluted for other serum proteins. We assessed the effect of albumin depletion on the activity of serum against *R. delemar* and another major human respiratory fungal pathogen *A. fumigatus*^7^. Interestingly, albumin depletion resulted in a significant loss of antifungal activity of the serum selectively against *R. delemar* but not against *A. fumigatus* (Fig. 1e).

Next, we purified human albumin from the sera of healthy volunteers to directly evaluate its antifungal activity against Mucorales via a protocol based on affinity column chromatography^21^ (**Extended Data Fig. 2a** and Fig. 1f). We found no evidence of transferrin, a serum iron transferring protein with important role in nutritional immunity against Mucorales^22^, in the albumin-containing eluted fractions by Western blot analysis (**Extended Data Figure 2b)**. Notably, purified human albumin dissolved at physiological concentrations (≈ 3.5 g/dL) in liquid culture media, had potent activity against *R. delemar* (Fig 1g). Additionally, purified albumin from different sources, including the commercially available bovine serum albumin (BSA) and human serum albumin (HSA) used for therapeutic applications, inhibited Mucorales growth when added at physiologically relevant concentrations in culture media (Fig 1h, i). Notably, we found that albumin specifically blocks lamentous (hyphal) growth of Mucorales, following the initial stage of isotropic growth (swelling) of fungal spores (**Extended Data Fig. 3a**). The antifungal activity of albumin was fungistatic, as it was fully reversible upon culture of inhibited Mucorales spores in fresh media without albumin (**Extended Data Fig. 3b**). Albumin selectively inhibited a wide range of clinical isolates of Mucorales species at physiological serum concentrations (4.5 g/dL), whereas it had no significant activity against other major human bacterial or fungal pathogens (Fig. 1j). Collectively, these results reveal the specialized activity of albumin against Mucorales and identify severe hypoalbuminemia as a biomarker of poor outcome of mucormycosis.

## The antifungal activity of albumin against Mucorales is mediated by the release of bound free fatty acids (FFAs).

To explore whether the inhibitory activity of albumin requires direct interaction of the protein with fungal cells, we filtered culture media containing inhibitory concentrations of albumin (4.5 g/dL) and assessed the antifungal activity of the culture filtrate (flow through). Notably, we found that the flow-through of albumin retains full inhibitory activity against *R. delemar* (**Extended Data Figure 4a**). Nutritional immunity via iron limitation is an essential host defense mechanism against Mucorales^1,15^. In view of the remarkable ability of albumin to bind a wide range of compounds, endogenous molecules, iron and other transition metals^23^, we explored the possibility that albumin inhibits Mucorales via depletion of essential nutrients from culture media. Therefore, we analyzed the components of regular RPMI culture media and found that the presence of albumin resulted in a significant reduction in the concentration of certain amino acids **(Extended Data Figure 4b and Supplementary Table 2**). However, supplementation experiments with amino acids and different combinations of nutrients contained in RPMI media did not affect the inhibitory activity of albumin flow-through against *R. delemar* (**Extended Data Figure 4c**).

We next considered the possibility that the release of an inhibitory molecule bound to albumin could account for the antifungal activity of the culture filtrate. Serum albumin acts as the main shuttle of non-esterified middle- and long-chain fatty acids (free fatty acids; FFAs) in extracellular uids^24^ and FFAs exert potent antimicrobial activity against microbial pathogens^25^. Therefore, we performed fractionation of lipid-containing elutions from a BSA-containing culture filtrate and functionally characterized the inhibitory activity against *R. delemar*. We identified a fraction with significant inhibitory activity against Mucorales, which contains high amounts of caprylic acid (C8:0) via GC-MS and ESI-HRMS analysis (**Extended Data Figure 5a, b and c**). We also confirmed that purified caprylic acid dissolved in ethanol (EtOH) has potent inhibitory activity against Mucorales at concentrations lower than those contained in the BSA filtrate **(Extended Data Figure 5d**).

**Figure 2:**
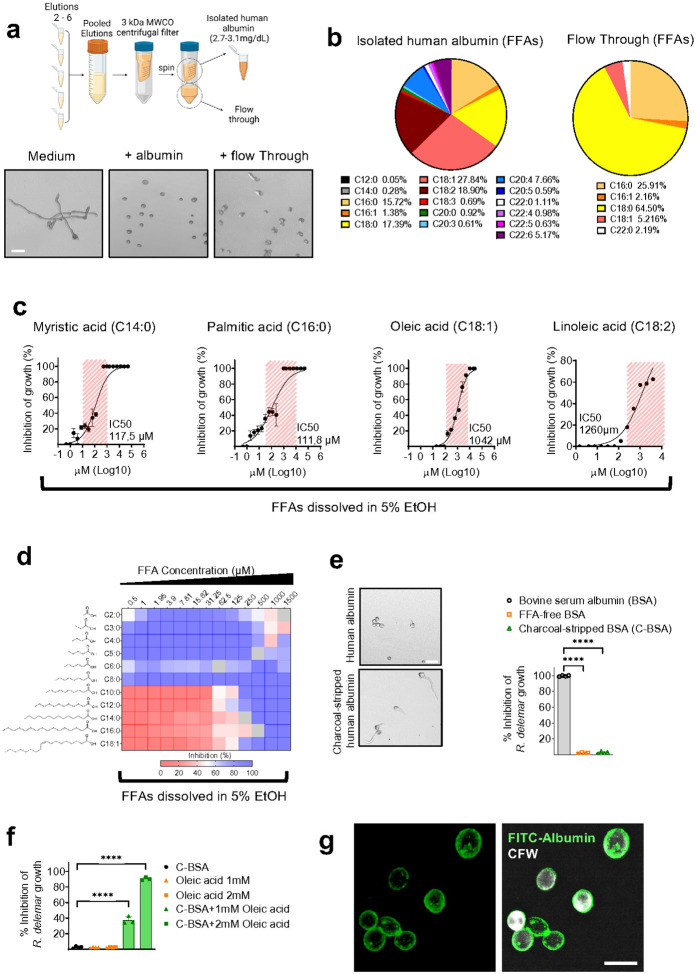
Physiological FFAs mediate the antifungal activity of albumin against Mucorales. **(a)** Outline of generation of flow-through and isolation of albumin from human serum following filtration with a 3 kDa molecular weight cut-off centrifugal filter (upper panel). Representative images of *R. delemar* spores cultured for 5 h in medium alone or medium supplemented with isolated human serum albumin or albumin flow through (lower panel). Scale bar, 50 mm. **(b)** Lipidomic analysis of FFAs of isolated human serum albumin and albumin flow through, obtained as described in **a**. **(c)** Antifungal activity of major serum FFAs against *R. delemar*. Red-striped areas designate physiological FFA serum concentrations. **(d)** Inhibitory effect of increasing concentrations of various short- to long-chain FFAs on *R. delemar* growth. **(e)**(Left) Representative images of *R. delemar* spores cultured for 5 h in mediasupplemented with isolated human serum albumin or charcoal-stripped isolated human albumin. Scale bar, 50 mm. (Right) Inhibitory effects of BSA, FFA-free BSA and charcoal-stripped BSA on *R. delemar* growth. ****p < 0.0001, one-way ANOVA and Tukey’s multiple comparisons post hoc test. **(f)** Inhibitory effects of charcoal-stripped BSA, oleic acid or charcoal-stripped BSA loaded with oleic acid on *R. delemar* growth. ****p < 0.0001, one-way ANOVA and Tukey’s multiple comparisons post hoc test. **(g)** Representative fluorescence images of CW-labelled *R. delemar* spores cultured for 5 h in the presence of FITC-labelled albumin. Scale. Scale bar, 20 mm.

To further explore the physiological relevance of our findings, we performed lipidomic profiling in purified human albumin before and after filtration. The albumin filtrate retained full inhibitory activity against Mucorales spores (Fig. 2a) and contained physiological middle-and long-chain FFAs (Fig. 2b and **Supplementary Table 3**). Furthermore, we found that purified serum FFAs dissolved in ethanol exerted potent anti-Mucorales activity at physiologically relevant concentrations (Fig. 2c). We also found that a broad range of FFAs of various carbon chain lengths and degrees of saturation display potent antifungal activity against Mucorales (Fig 2d). Importantly, purified human albumin following charcoal treatment for the removal of bound FFAs^26^, or commercially available BSA free of fatty acids (FFA-free BSA) had no activity against Mucorales (Fig. 2e). Furthermore, we found that charcoal treated BSA complexed with physiologically relevant concentrations of oleic acid (OA) dissolved the FFA and fully restored its activity against Mucorales (Fig. 2f). Experiments with fluorescent-labeled albumin further demonstrated that although albumin avidly binds to the fungal cell wall surface, it is not internalized by Mucorales spores (Fig. 2g). Collectively, these findings demonstrate that albumin binds, dissolves, shuttles and facilitates the release of physiological FFAs upon interaction with Mucorales to optimize their antifungal activity.

## Albumin-bound FFAs are protected from oxidation, which results in the loss of their inhibitory activity against Mucorales.

**Figure 3:**
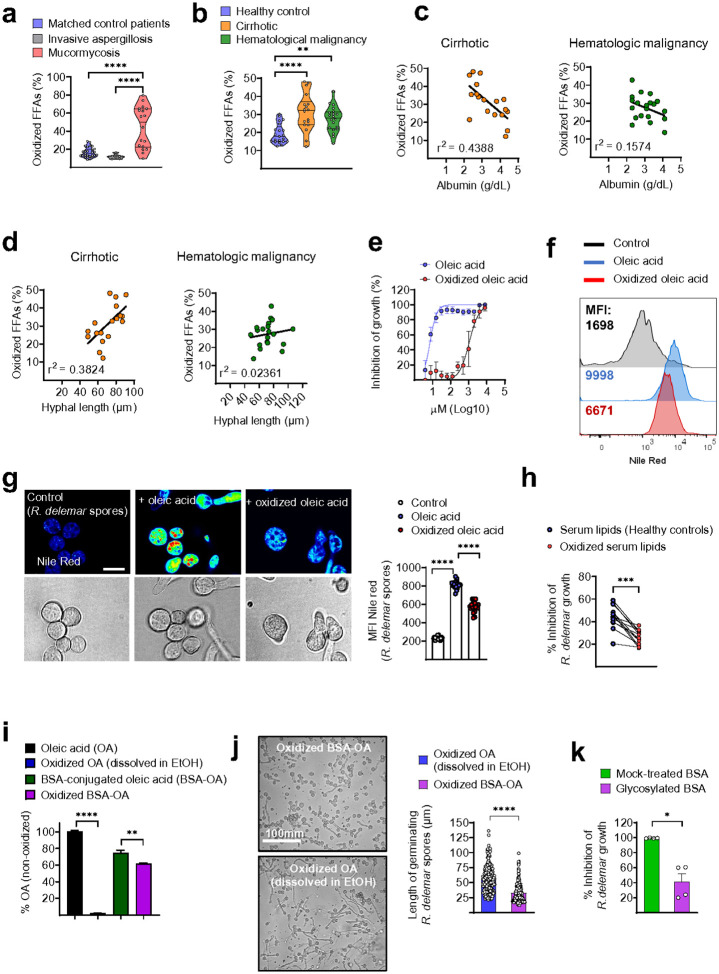
Albumin protects FFAs from oxidation and the subsequent loss of inhibitory activity against Mucorales. **(a)** Relative concentrations of oxidized FFAs analysed in the sera of matched controls (n = 27), patients with invasive aspergillosis (n = 6) and patients with mucormycosis (n = 18). ****p < 0.0001, one-way ANOVA and Tukey’s multiple comparisons post hoc test. **(b)** Relative concentrations of oxidized FFAs analysed in the serum of healthy controls (n = 21), cirrhotic patients (n = 18) and patients with hematologic malignancies (n = 20). **p = 0.0038, ****p < 0.0001, one-way ANOVA and Tukey’s multiple comparisons post hoc test. **(c, d)** Correlation of the percentage of oxidized serum FFAs with **(c)** albumin levels and **(d)** the inhibitory activity of sera from cirrhotic patients (n = 18) or patients with hematologic malignancies (n = 20) against *R. delemar*. A simple linear regression test was used to determine deviation from zero. **(e)**Inhibitory effects of increasing concentrations of oleic acid and oxidized oleic acid on *R. delemar* growth. **(f)** Representative flow cytometry analysis of Nile Red fluorescence intensity, which is indicative of FFA uptake by *R. delemar* spores cultured in medium, medium supplemented with oleic acid or medium supplemented with oxidized oleic acid **(g)**(Left) Representative fluorescent (upper panel) and bright field (lower panel) images of *R. delemar*spores stained with Nile Red as in f. Scale bar, 20 mm. (Right) Cumulative data of Nile Red mean fluorescence intensity (MFI) in *R. delemar* spores. ****p < 0.0001, one-way ANOVA and Tukey’s multiple comparisons post hoc test. **(h)** Serum lipids were isolated from healthy individuals and subjected to oxidation. The inhibitory effects of serum lipids and oxidized serum lipids on *R. delemar* growth were assessed (n = 13). ***p = 0.0002, Wilcoxon matched-pairs signed rank test. (i) GC–MS analysis of nonoxidized oleic acid (OA) content in OA and BSA-conjugated OA before and after microwave oxidation. **p = 0.0041, ****p < 0.0001, one-way ANOVA and Tukey’s multiple comparisons post hoc test. **(j)** Representative images of *R. delemar* spores cultured for 5 h in mediasupplemented with oxidized OA and oxidized BSA-conjugated OA (left panel). Scale bar, 100 mm. (Right) Length of germinating *R. delemar* spores cultured in mediasupplemented with oxidized OA and oxidized BSA-conjugated OA. ****p < 0.0001, Mann-Whitney test. **(k)** Inhibitory effect of mock-treated and glycosylated BSA on *R. delemar* growth. *p = 0.0286, Mann-Whitney test.

We next performed targeted lipidomic profiling of the sera of patients with mucormycosis and matched controls to analyze the FFA composition and identify abnormalities associated with the loss of antifungal activity in the serum. Interestingly, we found that the sera of patients with mucormycosis contained a significantly greater proportion of oxidized forms of FFAs than the sera of control patients, who were matched for the underlying disease without infection or those who have been diagnosed with invasive aspergillosis (Fig, 3a and **Supplementary Table 3**). Furthermore, the sera of patients susceptible to mucormycosis due to underlying cirrhosis or hematological malignancy, contained high amounts of oxidized FFAs, which was directly proportional to the severity of hypoalbuminemia (Fig. 3b, c). Importantly, the degree of FFA oxidation in patient sera strongly correlated with the loss of inhibitory activity against Mucorales (Fig. 3d). These findings suggested that oxidized FFAs display attenuated activity against Mucorales.

To directly evaluate the antifungal activity of oxidized FFAs we oxidized oleic acid (OA), a major physiological serum FFA, and assessed the effect on antifungal activity. We found that the oxidation of OA resulted in a > 100-fold decrease in its inhibitory activity against *R. delemar* (Fig. 3e). Because FFA oxidation results in reduced uptake by mammalian cells^27^, we evaluated whether the reduced antifungal activity of oxidized FFAs is related to diminished uptake by fungal cells. We used an established protocol to measure OA uptake by *R. delemar* cells via fluorescence labeling with Nile Red lipid dye^28^. Notably, we detected a significant degree of OA uptake by *R. delemar* spores within a few hours in culture, which was dramatically reduced upon OA oxidation (Fig 3f, g). To establish a causal relationship between FFA oxidation and the loss of anti-Mucorales activity in serum, we isolated serum lipids from healthy individuals and assessed the effects of oxidation on their antifungal properties. Importantly, oxidation of serum lipids resulted in a significant decrease in their inhibitory properties against *R. delemar* spores (Fig. 3h).

In view of the well-established antioxidant properties of albumin^23^, we reasoned that the binding of FFAs to albumin can protect them from oxidation. Therefore, we performed chemical oxidation of OA dissolved in EtOH or following complexation with albumin and assessed the degree of oxidation. We found that albumin significantly protected OA from oxidation (Fig. 3i) and retained its antifungal properties against Mucorales (Fig. 3j).

Albumin glycation is a characteristic abnormality in DM that results in the dissociation of FFAs from their binding sites and increased oxidation^29^. In view of the dominant role of DM as a predisposing factor for mucormycosis^1,2^, we performed *in vitro* glycation of albumin (BSA) and assessed its ability to inhibit Mucorales. Indeed, albumin glycation resulted in significant loss of albumin antifungal activity (Fig. 3k). Collectively, these studies reveal that albumin protects FFAs from oxidation, which results in the loss of their antifungal activity against Mucorales. Additionally, we identified FFA oxidation as a prominent abnormality in mucormycosis sera.

## Albumin-bound FFAs modulate Mucorales pathogenicity by targeting mucoricin expression.

**Figure 4:**
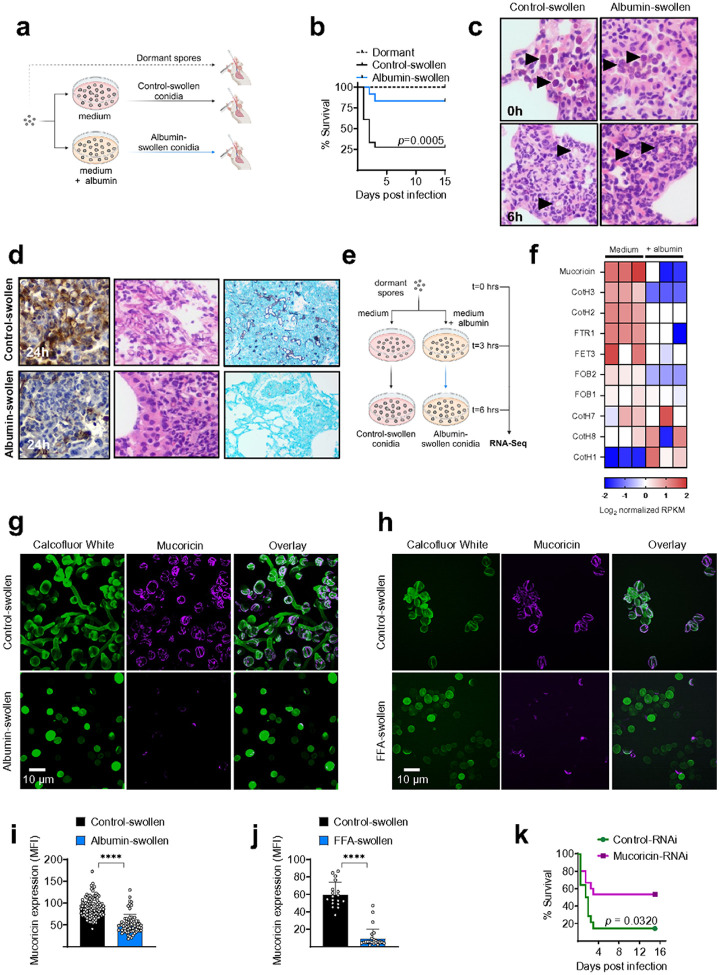
Albumin-bound FFAs target Mucorales pathogenicity by inhibiting the expression of mucoricin. **(a)** Outline of intratracheal (i.t.) instillation of dormant or swollen *R. delemar* spores. Swollen spores were generated via *R. delemar* culture in medium (control-swollen) or medium supplemented with 4.5 g/dL BSA (albumin-swollen) for 3 h. **(b)** Survival analysis of C57BL/6 mice infected i.t. with 2.5×10^6^ dormant (n = 6), control-swollen (n = 18) or albumin-swollen (n = 12) *R. delemar* spores as in a. The nonparametric log-rank (Mantel-Cox) test was used to calculate differences between survival curves. **(c)** Histopathology (H&E staining) of representative lung sections from mice at 0 h and 6 h post infection with control-swollen and albumin-swollen *R. delemar* spores as described in **a**. Black arrowheads designate fungal spores. Scale bar, 100 mm. **(d)** Histopathology (left: active caspase 3; middle: H&E; right: GMS staining) of representative lung sections from mice on day 1 post infection with control-swollen (upper panel) and albumin-swollen (lower panel) *R. delemar* spores as in **a**. Scale bar, 100 mm. **(e)** Outline of RNA-sequencing (RNA-seq) analysis of dormant and swollen *R. delemar* spores. Swollen spores were generated via *R. delemar* culture in medium (control-swollen) or medium supplemented with BSA(albumin-swollen) for 3 h and 6 h. **(f)** Differential mRNA expression of *R. delemar* virulence-related genes following incubation in medium containing albumin (+albumin) or without albumin (medium) for 6 hours. The log (base 2)-transformed RPKM values that were normalized across all 6 samples are plotted. Red indicates high gene expression; blue indicates low expression. Each column represents an individual sample. **(g)** Representative confocal images of mucoricin expression in CFW-labelled, control- andalbumin-swollen *R. delemar* spores after 3 h of culture. Scale bar, 10 mm. **(h)** Representative confocalimages of mucoricin expression in CFW-labelled, control- and FFA-swollen *R. delemar* spores. Scale bar, 10 mm. **(i)** Cumulative data of mucoricin expression in control and albumin-swollen *R. delemar* sporesacquired in **g**. ****p < 0.0001, Mann-Whitney test. **(j)** Cumulative data of mucoricin expression in control- and FFA-swollen *R. delemar* spores acquired in **h**. ****p < 0.0001, Mann-Whitney test. **(k)** Survivalanalysis of C57BL/6 mice infected i.t. with 2.5×10^6^ swollen control-RNAi (n = 14) or mucoricin-RNAi (n = 15) *R. delemar* spores. The nonparametric log-rank (Mantel-Cox) test was used to calculate differencesbetween survival curves.

We next investigated the physiological importance of albumin during *in vivo* infection with Mucorales, using an established model of pulmonary mucormycosis in immunocompetent mice infected with swollen fungal spores^15^. Specifically, we allowed dormant spores of *R. delemar* to grow in culture media with or without physiological concentrations (4.5 g/dL) of albumin for ≈ 4 hours and performed intratracheal infection of the mice. We found that albumin preexposure rendered *R. delemar* spores almost completely avirulent *in vivo* (Fig. 4a, b). Interestingly, pre-exposure to albumin did not inhibit the *in vivo* germination of Mucorales during the early stages of infection in the lungs (Fig. 4c). Instead, albumin preexposure completely abrogated massive tissue necrosis and tissue invasion induced by germinating fungal spores, as evidenced by staining for active caspase 3 in histopathology sections of the lung (Fig. 4d). These findings suggest a predominant effect of albumin in attenuating the virulence properties of germinating Mucorales spores.

Next, we performed RNA-seq analysis during the *in vitro* growth of *R. delemar* to explore the molecular mechanism of action of albumin on the pathogenicity program of the fungus. We Focused on the transcriptional response of all characterized virulence factors of Mucorales, including CotH invasins^1^, the mycotoxin mucoricin^10^, and genes regulating the iron assimilation program of the fungus^1^. Notably, we identified mucoricin as the gene most significantly downregulated by albumin (Fig 4e, f). We also found that preexposure of Mucorales to the albumin culture filtrate almost completely abolished the expression of mucoricin on the surface of swollen spores, as evidenced by immunostaining (Fig. 4g, i); similarly, exposure of *R. delemar* spores to purified FFAs blocked mucoricin protein expression (Fig 4h, j). Finally, the silencing of mucoricin in *R. delemar* (mucoricin RNAi strain^10^) resulted in a significant decrease in the pathogenicity of swollen spores following pulmonary infection of immunocompetent mice (Fig. 4k). Overall, these results identify mucoricin as a molecular target of albumin-bound FFAs during *in vivo* fungal growth.

## Albumin knockout mice display selective susceptibility to mucormycosis.

**Figure 5:**
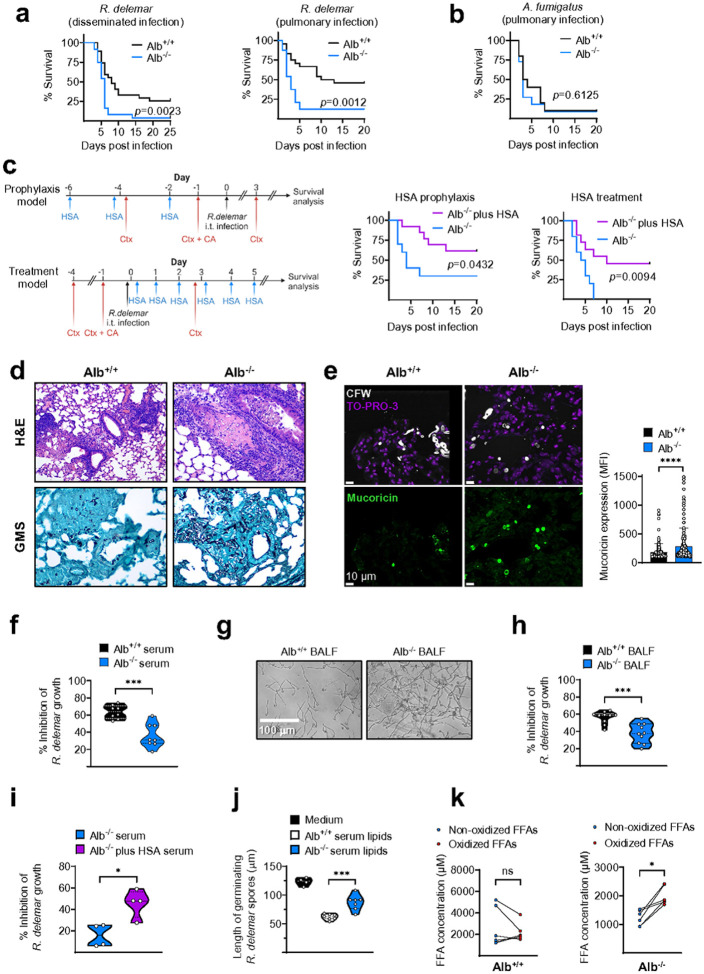
Albumin knockout (KO) mice display selective susceptibility to mucormycosis. **(a)** (Left panel) Survival analysis of WT (Alb^+/+^) and Albumin-KO (Alb^−/−^) mice infected intravenously with 1×10^5^ dormant *R. delemar* spores (disseminated infection, n = 24–27). (Right panel) Survival analysis of neutropenic Alb^+/+^ and Alb^−/−^ mice infected i.t. with 1×106 dormant *R. delemar* spores (pulmonary infection, n = 24). **(b)** Survival analysis of neutropenic Alb^+/+^ and Alb^−/−^ mice infected i.t. with 1×10^6^ dormant *A. fumigatus* spores (pulmonary infection, n = 11). **(c)** (Left panel) Outline of prophylactic and therapeutic administration models of FFA-free HSA in pulmonary mucormycosis. (Right panel) Survival analysis of neutropenic Alb^−/−^ mice, either untreated or administered FFA-free HSA prior to infection (left; prophylactic model, n = 10–13) or upon i.t. infection (right; therapeutic model, n = 10–11) with 1×10^6^ dormant *R. delemar* spores. The nonparametric log-rank (Mantel-Cox) test was used to calculate differences between survival curves in **a-c**. **(d)** Histopathology (upper panel: H&E; lower panel: GMS staining) of representative lung sections from neutropenic Alb^+/+^ and Alb^−/−^ mice on day 3 of infection with *R. delemar* spores. Scale bar, 100 mm. **(e)** (Left panel) Representative fluorescence images of mucoricin expression in lung sections from neutropenic Alb^+/+^ and Alb^−/−^ mice on day 3 of infection with *R. delemar* spores. Scale bar, 100 mm. (Right panel) Cumulative data of mucoricin expression in lung sections from neutropenic Alb^+/+^ and Alb^−/−^ mice on day 3 of infection with *R. delemar* spores. ****p < 0.0001, Mann-Whitney test. **(f)** Inhibitory effect of mouse serum from Alb^+/+^ and Alb+ mice on *R. delemar* growth (n = 8). ***p = 0.0006, Mann-Whitney test. **(g)** Representative images of *R. delemar* spores cultured for 5 h in BALF from Alb^+/+^ and Alb^−/−^ mice. **(h)** Inhibitory effect of mouse BALF obtained from Alb^+/+^ and Alb^−/−^ mice on *R. delemar* growth (n = 8). ***p = 0.0007, Mann-Whitney test. **(i)** Inhibitory effect of mouse serum from Alb^−/−^ mice and Alb^−/−^ mice prophylactically supplemented with FFA-free HSA, as in **c**, on *R. delemar* growth (n = 4). *p = 0.0286, Mann-Whitney test. **(j)** Inhibitory effect of isolated mouse serum lipids from Alb^+/+^ and Alb^−/−^ mice on *R. delemar* growth (n = 7). ***p = 0.0005, one-way ANOVA and Tukey’s multiple comparisons post hoc test. **(k)** Concentrations of non-oxidized and oxidized FFAs in the serum of Alb^+/+^ (left panel) and Alb^−/−^ (right panel) mice (n = 6). *p = 0.0313, Wilcoxon matched-pairs signed rank test.

We next employed a humanized model of albumin knockout (KO) transgenic mice to genetically validate the role of albumin in host defense against Mucorales^30^. These transgenic mice are double KO for albumin and the neonatal Fc receptor (FcRn), which regulates the recycling of albumin, and transgenic for human FcRn. The expression of human FcRn results in a prolonged half-life of human albumin following systemic administration^30^. We found that albumin KO (Alb^−/−^) mice were highly susceptible to disseminated infection with *R. delemar* following intravenous injection of fungal spores (Fig. 5a). Additionally, pulmonary infection of Alb^−/−^ mice with *R. delemar* resulted in significantly higher mortality rates than infection of control Alb^+/+^ mice in a pathophysiologically relevant neutropenic model of pulmonary mucormycosis^31^ (Fig. 5a). However, Alb^−/−^ mice displayed comparable susceptibility to control Alb^+/+^ mice following infection with *A. fumigatus* in the neutropenic model of invasive aspergillosis (Fig. 5b). Importantly, prophylactic or therapeutic administration of purified human albumin fully restored the resistance of Alb^−/−^ mice to pulmonary mucormycosis to a comparable degree to that of Alb^+/+^ mice (Fig. 5c). Histopathological sections of lungs from neutropenic Alb^−/−^ vs. Alb^+/+^ mice following pulmonary infection with *R. delemar* revealed invasive fungal growth (Fig. 5d) and significantly higher expression of mucoricin on germinating hyphae as evidenced by immunostaining of the tissue (Fig. 5e). Furthermore, sera (Fig. 5f) and bronchoalveolar fluid (BALF) (Fig. 5g, h) obtained from Alb^−/−^ mice exhibited significant loss of inhibitory activity against *R. delemar* growth, which was reversed upon systemic albumin administration (Fig. 5i). Accordingly, serum lipids isolated from Alb^−/−^ mice had diminished inhibitory activity against Mucorales compared with the serum lipids of control Alb^+/+^ mice (Fig. 5j). Lipidomic analyses of FFAs in sera revealed a significantly higher proportion of oxidized FFAs in Alb^−/−^ than in Alb^+/+^ mice (Fig. 5k and **Supplementary Table 3**). These results provide definitive evidence of the master regulatory role of albumin in host defense against Mucorales, and justify future evaluation of its clinical use in the management of mucormycosis.

## Discussion

Multiple lines of evidence suggest that mucormycosis occurs upon failure of uncharacterized metabolic host defense mechanisms to inhibit the growth of inhaled fungal spores. First, mucormycosis predominantly affects patients with a broad range of seemingly unrelated metabolic abnormalities^1,2,4^ in the absence of primary immune dysfunction. Second, congenital immunodeficiencies associated with a unique predisposition for mucormycosis have not been identified^32^. Accordingly, mucormycosis is a relatively uncommon disease in severely immunocompromised patients with prolonged pancytopenia. Third, prompt reversal of the underlying metabolic abnormalities markedly improves mucormycosis outcome^1^. Fourth, nutritional immunity via iron sequestration has an essential role in control of Mucorales infection, from *Drosophila* to humans^1,15,33,34^. On the other hand, the saprophytic nature of Mucorales in mammals, mandates the evolution of specialized metabolic host factors that block fungal pathogenicity *in vivo*.

In this study, we discovered a non-redundant role of albumin in anti-Mucorales host defense and delineated the underlying molecular mechanism. Our initial studies revealed an intriguing association between low albumin level and the development of mucormycosis in high-risk patients with hematological malignancies. Furthermore, severe hypoalbuminemia was a predictor of poor outcome in independent cohorts of patients with mucormycosis, on three different continents. These compelling findings are in line with previous reports on the increased susceptibility of patients with disease-related malnutrition to mucormycosis^1,2,5,19,35,36^. Given the lack of host biomarkers for prognostication of mucormycosis, albumin level should be further investigated as a readily available, inexpensive and universal biomarker to stratify patients at risk and predict infection outcome.

The striking correlation between albumin level and the antifungal activity of serum from patients at risk for mucormycosis, prompted us to evaluate the direct inhibitory effect of albumin on Mucorales. Previous studies reported discordant results on the *in vitro* antimicrobial properties of albumin, without insight into the underlying mechanism and its physiological importance during infection^37–40^. Our experiments following human serum albumin depletion and purification, along with susceptibility studies across a range of human pathogens, provided unambiguous evidence of the specialized inhibitory activity of albumin against Mucorales.

Nutritional immunity via iron limitation inhibits Mucorales growth, both extracellularly and following phagocytosis by AMs^1,15,22^. Furthermore, increased iron availability due to iron overload or acidosis is an important pathogenic mechanism in mucormycosis^22,41^. Systematic analysis of the pH and composition of albumin containing media before and after filtration, and susceptibility studies following nutrient supplementation excluded the possibility of an underlying nutritional immunity mechanism involved in the albumin inhibition of Mucorales. In contrast, functional analysis of lipid-containing fractions and lipidomic profiling of the albumin culture filtrate revealed that the antifungal activity of albumin is exclusively mediated by the release of bound physiological FFAs. Notably, the antimicrobial and immunomodulatory properties of naturally occurring FFAs have long been recognized^25,42,43^. Previous studies on the antimicrobial properties of host FFAs Focused on the epithelial surfaces of the skin and mucosal surfaces, where FFAs can reach concentrations that are orders of magnitude greater than those in serum and other extracellular uids^43–46^. Importantly, the host defense property of albumin-bound FFAs has not been previously demonstrated. Instead, the scavenging of albumin-bound FFAs is a virulence strategy that promotes the growth of bacterial pathogens^47^, which is in line with our findings concerning the lack of antimicrobial activity of albumin against human pathogens other than Mucorales.

Further experiments demonstrated the essential role of albumin in binding, dissolving, shuttling and protecting FFAs from oxidation. Collectively, these unique properties of albumin optimize FFA accumulation inside Mucorales spores and result in the inhibition of fungal growth. Additionally, the high degree of FFA oxidation in the sera of mucormycosis patients is associated with the loss of antifungal activity, a finding that further illustrates the physiological importance of albumin in host defense against Mucorales. Fungal oxylipins are enzymatically oxidized forms of FFAs with important signaling properties for lamentous growth and virulence of another major human fungal pathogen, *A. fumigatus*^48^. Whether certain serum oxylipins enhance fungal growth and promote the pathogenicity of Mucorales deserves investigation in future studies. Finaly, we found that the glycation of albumin, a modification associated with the loss of FFA-binding capacity in patients with poorly controlled DM^29^, resulted in a significant loss of its inhibitory activity against Mucorales. Collectively, these findings identify a specialized role of albumin-bound FFAs in immunity against Mucorales with important implications in the pathogenesis of mucormycosis.

To gain mechanistic insight into the physiological importance of albumin in antifungal immunity we employed pathophysiologically relevant *in vivo* models of mucormycosis in immunocompetent and neutropenic mice. Notably, preexposure to albumin almost completely abrogated virulence of germinating *R. delemar* spores during *in vivo* infection of immunocompetent mice, as a result of the transcriptional inhibition of the expression of mucoricin, a potent mycotoxin that was previously reported to be critical for disease progression and is required for the tissue necrosis commonly associated with invasive mucormycosis^11^. The robust inhibitory effect of albumin on mucoricin expression was fully mediated by FFAs and was further validated at the protein level. Pulmonary infection with germinating spores of a mucoricin RNAi *R. delemar* strain confirmed the major pathogenetic role of this mycotoxin in the development of mucormycosis in immunocompetent mice. Interestingly, FFAs target the virulence properties of human bacterial pathogens via a broad range of strategies, including the inhibition of transcription^42,46,49,50^. Future work will delineate the precise molecular mechanism of the inhibition of mucoricin expression by FFAs.

Studies in mouse models of disseminated mucormycosis and pulmonary fungal infection by *A. fumigatus* and Mucorales in albumin-KO humanized mice provided additional definitive evidence for a non-redundant, specialized role of albumin in immunity against Mucorales. These studies are in concordance with results of human cohorts of mucormycosis, by demonstrating a significant loss of antifungal activity of serum and BAL fluid against Mucorales and the presence of high amounts of oxidized FFAs in the sera of albumin KO mice. Finally, the reversal of susceptibility of albumin KO mice to Mucorales infection upon albumin supplementation, provides a strong rationale for the correction of hypoalbuminemia as (a) a low-cost preventive strategy in high-risk patients and (b) an adjunct therapeutic modality in established mucormycosis. The precise therapeutic role of albumin in the management of mucormycosis should be further evaluated in prospective clinical studies.

In this study, we identified a novel metabolic host defense mechanism that specifically targets the pathogenicity program of Mucorales and is regulated by albumin. Furthermore, our findings lead to a new pathogenetic model of mucormycosis (**Extended Data Figure 6**), which provides mechanistic insight into the unique epidemiological features of this disease. Physiologically, albumin-bound FFAs prevent invasive growth of constantly inhaled Mucorales spores by inhibiting lamentous growth and targeting the expression of the newly identified mycotoxin mucoricin. Albumin solubilizes, transports, and protects bound FFAs from oxidation, thus potentiating their activity against Mucorales in serum and the extracellular space. Metabolic abnormalities that result in severe hypoalbuminemia or chemical modifications that impair binding capacity of the protein for FFAs (e.g. glycation), lead to increased FFA oxidation and loss of their antifungal activity against Mucorales. Additionally, systemic oxidative stress induced by hyperinflammation, underlying comorbidities, or iron overload could further promote FFA oxidation and increase susceptibility to mucormycosis^1^.

Finally, our work provides interesting cues on the mechanisms of selection pressure that account for the enigmatic evolution of albumin in vertebrates^51^, by linking its unique lipid binding properties with the modulation of the pathogenicity of ubiquitous, toxin-producing phytopathogenic fungi. Collectively, our findings have profound implications for the immunopathogenesis and therapeutics of an emerging human fungal disease with limited treatment options.

## Materials And Methods

### **Microorganisms and culture conditions**.

*Rhizopus delemar* (99–880)^10^, *R. arrhizus* (557969)^34^, and *Cunninghamella bertholletiae* (506313)^34^, have been previously described. *Mucor circinelloides* (JMRC:NRZ:0774), R. *pussilus* (JMRC:NRZ:0496), *R. microsporus* (JMRC:NRZ:0680), *A. fumigatus* (ATCC 46645), *A. flavus* (JMRC:NRZ:0756), *A. terreus* (JMRC:NRZ:0442), *C. albicans* (JMRC:NRZ:1000), *C. glabrata* (JMRC:NRZ:1006), and *F. proliferatum* (JMRC:NRZ:0657) were obtained from the Leibniz Institute for Natural Product Research and Infection Biology, Hans Knöll Institute, Jena, Germany. All Mucorales isolates were cultured on potato dextrose agar (PDA; Becton Dickinson) plates for 7 days at 37°C. *R. delemar* M16 is a previously described pyrf-null mutant derived from *R. delemar* 99–880 that is unable to synthetize uracil^52^. *R. delemar* transformed with RNA interference (RNAi) targeting mucoricin expression and *R. delemar* transformed with empty plasmid (control) were derived from strain M16, as previously described^10^. For the experiments involving these RNAi strains, a synthetic medium containing yeast nitrogen base (YNB, BD) supplemented with a complete supplemental mixture without uracil (CSM-URA, MP Bi°Chemicals) (i.e. YNB+CSM-URA) was used.

All the bacterial isolates used (*Escherichia coli, Pseudomonas aeruginosa, Klebsiella pneumoniae*, and Staphylococc*us aureus*) were clinical isolates obtained from the University Hospital of Heraklion, Crete. All bacteria were streaked onto plates containing LB agar plates from a freshly prepared frozen glycerol stock. Upon growth on LB agar plates, single colonies were used to inoculate overnight cultures of LB (50 mL at 37°C).

The next day, a volume of 1 mL of each of these cultures was diluted 1:10 to a final volume of 10 mL in LB media and incubated in conical asks for 2 h, at 37°C, with constant shaking, to reach mid-log phase of growth. A volume of 200 μL was taken from each culture and the optical density (OD) was measured at 600 nm. The desired volume from each culture was used and centrifuged at 2000 rpm for 2–3 min. Bacteria pellets were washed 3 times with LB media. Bacteria pellets were diluted in RPMI media or RPMI plus 4.5g/dL of albumin to the desired OD = 0.2. A volume of 200μL from each test condition was transferred to a flat bottom 96 well plate. The plates were incubated at 37°C, with constant shaking. At regular time intervals (45 min), 10 μL from each culture condition was diluted in RPMI media to a final volume of 100 μL and the OD600 was measured spectrophotometrically. The mean value of triplicate measurements of bacterial growth in regular media at t = 340 min was defined as 100% growth.

### Human albumin depletion and purification.

Albumin was depleted from human serum via Cibacron Blue 3G-A beads (Albumin Depletion Kit, Abcam) according to the manufacturer’s instructions. The Blue Sepharose 6 Fast Flow (GE Healthcare) was first rehydrated with an albumin-free serum filtrate (generated through serum filtration via Amicon 50 kDa molecular weight cut-off ultracentrifugal filters; Merck), incubated with fresh human serum (3 mL) at 4°C O/N with rotation and then packed back in a column. The first volume (3 mL) of the flow through contained albumin depleted human serum. The column was subsequently washed with 7 mL of wash buffer (20 mM Na_2_HPO_4,_ 20 mM NaH_2_PO_4,_ pH 8). Albumin was isolated from the column in 6 consecutive elutions using 7 mL of elution buffer (2M NaCl, 20mM Na_2_HPO_4_, pH 8) each time. Elution fractions 2–6 were pooled and further processed for *in vitro* experiments.

Owing to the increased amount of NaCl in the eluted fractions, dialysis was performed using a CelluSep T2 membrane (Orange Scientific, Cellusep T2 Tubings, 6000–8000MWCO), which was embedded in the appropriate buffer (20 mM Na_2_HPO_4,_ 20 mM NaH_2_PO_4,_ pH 8) for 4 h at 4°C to achieve a physiologically relevant NaCl concentration (150 mM). Elutions were then condensed using Amicon 3kD MWCO ultracentrifugal filters (Merck), to a final volume of 2 mL and filter sterilized through 0.22 μm Spin-X centrifuge tube filters (Costar).

### Chemical modifications of albumin.

Highly oxidized albumin was prepared by the incorporation of cysteine into reduced albumin^53^. BSA was treated with a 50-fold molar excess of L-cysteine/cystine by mixing 80 mL of 0.06 mM BSA with 72 mL of 3 mM cysteine and 8 mL of 3 mM cystine. All the solutions were diluted in 0.1 M sodium carbonate and hydrogen carbonate buffer (pH 10.0), and upon incubation at 37°C for 48 h, the resulting mixture was lyophilized. Next, the residue was dissolved in PBS and purified from low molecular weight components (excess cysteine/cystine) by filtration through an Ultrafree-3000 Da membrane (Millipore) at 4°C. Purified, highly oxidized albumin was lyophilized and stored at RT. Glycosylated albumin was prepared by diluting albumin in PBS containing 5 mM glucose^54^. The solution was incubated for 72 h under an atmosphere of 95% O_2_, and 5% CO_2_ to maintain the pH at 7.3 – 7.4. The mixture was then lyophilized, followed by solubilization in PBS and purification through ultrafiltration with an Ultrafree-3000 Da membrane. Purified, glycosylated albumin was lyophilized and stored at RT. The control solutions were prepared in PBS and subjected to the same lyophilization and filtration processes to increase the reliability of the results. The dried, equal weight samples were derivatized with 20 μL of 20 mg/mL methoxy amine/pyridine and 50 μL of hexane. The mixture was vortexed and kept at 37°C for 1.5 h, with vortexing every 30 min^93,94^. Ninety microliters of (N-trimethylsilyl-N-methyl triluoro-acetamide and trimethyl-chlorosilane (MSTFA + 1% TMS) were added and the samples were vortexed for 30 sec and incubated at 37°C for 1 h. The obtained solution was filtered through 0.45 mm syringe filters (nylon syringe filter, Membrane Solutions, Auburn, WA, USA) and subjected to GC-MS/MS in multiple reaction monitoring (MRM) mode analysis according to previously published methods^55,56^. The obtained metabolites were imported into MetaboAnalyst (v.5.0, https://www.metaboanalyst.ca/home.xhtml). Hierarchical cluster analysis (HCA) and partial least squares-discriminant analysis (PLS-DA) were then performed according to previous methods^57^.

The removal of FFAs from albumin (BSA and HSA isolated from donors) was performed with the use of activated charcoal, as previously described with some modifications^26^. Next, activated charcoal and albumin were mixed at a ratio of 1:2 for 1 h at 4°C. The excess of activated charcoal was removed by ultracentrifugation at 20,200 × g for 30 min at 4°C.

Loading of albumin with FFAs was performed as previously described^58,59^ with some modifications. Brie y, 4.5 g/dL charcoal-treated albumin (diluted in RPMI medium) and 80 mM FFAs (diluted in ethanol) were heated at 55°C for 30 min and 5 min, respectively. Then, FFAs and albumin were gently mixed at a ratio of 8:1. The FFA-albumin mixture was thoroughly mixed by vortexing and incubated at 37°C for 1 h. Effective conjugation of FFAs to albumin resulted in a clear solution.

### *In vitro* assessment of the inhibitory activity of albumin and human serum.

Fungal conidia (spores) were harvested by gentle shaking in the presence of sterile 0.1% Tween 20 in PBS, washed twice with PBS, filtered through a 40-μm-pore-size cell strainer (Falcon) to separate conidia from contaminating mycelium, counted by a hemocytometer, and suspended at a concentration of 10^8^ spores/mL. To achieve synchronized swelling of *Rhizopus* conidia, 1×10^6^/mL dormant conidia were incubated for 4–6 h at 28°C in a 6-well plate in RPMI-MOPS (pH 7,0) supplemented with 2% glucose +/− 4.5 g/dL BSA or other sources of albumin. To study the minimal growth requirements of *R. arrhizus (*a minimal-growth-requirements medium was used^60^ based on HBSS supplemented with 0.05% w/v MgSO_4_-7H_2_O, 0,1% w/v glucose and 0,1% w/v NH_4_Cl. For fluorescence labeling, the spores were stained with 20 μg/mL Fluorescent Brightener 28 (Calcofluor White, Sigma Aldrich) in 0,1 M NaHCO_3_ pH 8.3 at room temperature (RT) in the dark for 1 h, with constant rotation.

In another set of experiments, *R. delemar* spores were cultured at 37°C in RPMI-MOPS without phenol red adjusted to pH 7, supplemented with 4.5 g/dL BSA and 450 μg/mL FITC-albumin (Sigma Aldrich) in a 96-well μ-Plate (Ibidi). After 5 h of culture *R. delemar* live spores were imaged with a spinning disk confocal microscope (Dragon y 200, Andor).

To assess the inhibitory activity of human serum, 1 × 10^4^ fungal spores were incubated in at bottom 96 well plates containing 100 μl of serum from healthy individuals or patients at 37°C and 5% CO2 for 5–6 h. At least three different fields of each well (a total of >100 germinating spores) were imaged under an inverted microscope (Olympus) and the length of the germinating conidia was measured using ImageJ^61^. In certain experiments, time lapse videos of fungal growth were acquired with the use of an Operetta high screening content system (Perkin Elmer) at room temperature with 5% CO_2_. Photographs of each well were automatically taken approximately every 30 min for 18 timepoints and analyzed with *Harmony4.1* software(Perkin Elmer).

The inhibition of fungal growth was additionally quantitated via an XTT metabolic activity assay (Biotium). A solution of XTT tetrazolium salt (0.25 mg/mL) and menadione (25 μM) was freshly prepared in PBS and added to the cell culture. Fungal spores were further incubated at 37°C for another 1 hour^38^. Fungal spore-free culture supernatants were collected, and their absorbances at 450 nm (OD450) and 620 nm (OD620) were measured via a microplate photometer (Multiscan, Thermo). Growth inhibition was calculated according to the following formula:

$$\text{Growth}\;\text{Inhibition}\;(\%)=100*\frac{\left(\text{OD}450-\text{OD}620\right)\;\text{control}\;\text{cells}-\left(\text{OD}450-\text{OD}620\right)\;\text{treated}\;\text{cells}}{\left(\text{OD}450-\text{OD}620\right)\;\text{control}\;\text{cells}}$$

### *In vitro* studies on the antimicrobial activity of FFAs.

FFA stock solutions were initially dissolved in absolute ethanol (EtOH) to a final concentration of 80 mM and incubated at 95°C for 5 min. 2X MOPS-RPMI media was also heated at 95°C for 30 min and serial dilutions of FFAs were made by slowly adding the desired volume of preheated FFAs into the medium. The final concentration of EtOH used under culture conditions was 5% to ensure optimal dilution of FFAs; we confirmed that 5% EtOH had no significant inhibitory effect on Mucorales growth. Fungal spores were incubated in FFA-containing MOPS-RPMI medium at 37°C for 16–18 h and fungal germination was measured as previously described.

### Lipid extraction.

Lipids were extracted via a CHCl3:CH3OH (2:l) solution according to a previously published method^62^, dried with anhydrous sodium sulfate, subjected to residual evaporation via a rotatory evaporator (Buchi) under an inert stream of nitrogen and then stored at − 20°C ^91^. The residue obtained was equally divided into two parts; one for the oxidation reaction and the other left under inert conditions to avoid auto-oxidation.

### Microwave-assisted oxidation of FFAs.

FFAs and BSA-bound FFAs were subjected to oxidation via an irradiation microwave reactor following modified previously published methods^92^. The samples were dissolved in 0.2 mM H_2_O_2_ in methanol to a final volume of 10 mL. The oxidization reaction was performed in a sealed vial on a synergy microwave synthesizer (CEM) for 20 min at an operating temperature of 100°C and a pressure of 200 psi. The reaction mixture was extracted with CHCl3, dried with anhydrous sodium sulfate, subjected to residual evaporation via a rotatory evaporator (Buchi), and stored at −20°C.

### **Open air-assisted oxidation of FFAs**.

FFAs were diluted in absolute ethanol to a final concentration of 80 mM and exposed to open air inside a laminar flow hood, as previously described^63^. After 4 days the ethanol was evaporated, and the precipitates of the oxidized FFAs were rediluted in 0.01 M Tris-HCl (pH 7.5) and stored at −20°C.

### Human clinical studies.

The medical records of contemporaneous patients who had been admitted at the Leukemia Department of the MD Anderson Cancer Center were retrospectively reviewed. Standardized EORTC/MSG criteria were applied for diagnosis of pulmonary mucormycosis and pulmonary aspergillosis^64^ Clinical information of matched control patients for the underlying disease who developed bacterial (*Legionella*) pneumonia were also reviewed. Albumin levels were retrieved from the medical records of all the patients on the day of hospital admission. Information on demographics, underlying disease and risk factors for mucormycosis was collected. The study was approved by the IRB (IRB Protocol: PA14–0802) of the MDACC.

The medical records of consecutive patients admitted to the Department of Pulmonary Medicine, Department of Medical Microbiology, Institute of Medical Education and Research (PGIMER), Sector-12, Chandigarh, 160012, India over the past 8 years (2016–2023) with a diagnosis of pulmonary mucormycosis were retrospectively evaluated. The study was approved by the IRB (IEC-INT/2023/Study-1564).

Serum samples were prospectively collected from healthy individuals, cirrhotic patients, and patients with hematological malignancies at the University Hospital of Heraklion, Crete, and the Therapeutics Clinic of the General Hospital of Athens “Alexandra”. Demographics, clinical characteristics and serum albumin level, total free fatty acid (FFA) and oxidized FFA levels were measured in the sera of healthy individuals and patients. Approval for the collection of clinical information and blood samples from all individuals mentioned above was obtained from the Ethics Committee of the University of Heraklion, Crete, Greece (5159/2014, 10925/201 and 13-04-22/7970) and Therapeutics Clinic (122/18-2-2021). All patients were fully informed and provided written informed consent in accordance with the Declaration of Helsinki.

For lipidomic and functional studies, serum samples from patients with mucormycosis, or invasive pulmonary aspergillosis and matched control patients were obtained from hematological malignancy patients admitted to the University Hospitals of Leuven, Belgium (IRB# S61797).

### **Vitamin quantification in culture media via LC-MS**.

Samples were first filtered through 0.22 μm nylon syringe filters before undergoing LC-MS/MS analysis. The analysis was performed via an Elute UHPLC system linked to a Q-TOF mass spectrometer. Chromatographic separation was performed on a Hamilton^®^ Intensity Solo C18 column, maintained at 35°C. The sample mixure was 10 μL in a solvent system composed of 0.1% formic acid in deionized water (solvent A) and acetonitrile (solvent B). The elution gradient progressed from 1% to 99% solvent B over 20 min, with flow rates adjusted between 0.25 mL/min and 0.35 mL/min. Vitamin detection followed established methods by employing standards of cyanocobalamin (B12), folic acid (B9), riboflavin (B2), thiamine (B1), biotin (B7), Ca-panthenoate (B5), pyridoxine (B6), nicotinamide (B3), choline chloride, and PABA, all sourced from Sigma-Aldrich. Stock solutions of 5 mM for vitamins B1, B3, B5, B6, B7, B9, and B12, and choline chloride were prepared in water, with riboflavin (B2) in DMSO. Internal standard solutions were made at 10 mM, and working solutions comprised 200 mM of all vitamins in water and 100 mM of internal standards. Calibration curves were prepared using 0.1% formic acid in water, spanning six serial dilutions from 0 to 100 mM, each including 2.5 mM internal standards. Samples and calibration solutions underwent identical processing involving liquid-liquid extraction and drying prior to analysis, ensuring precise quantification of vitamins with detection limits in the low μg/L range. Each sample was injected three times.

### Analysis of trace elements in culture media using ICP-MS.

Inductive Coupled Plasma Mass Spectrometry (ICP-MS-9800 Series, USA) was used to detect trace metals in the samples. The samples were collected and digested with hydrochloric acid, to ensure complete dissolution. Microwave-assisted digestion was used for rapid and efficient sample preparation. The digested samples were then diluted with deionized water such that the concentration was within the measurable range of the ICP-MS instrument. Calibration standards with known concentrations of Na, K, Ca, Mg, Cu, and Fe were prepared, and isotope-labeled internal standards were used to correct for matrix effects and signal fluctuations. The prepared samples were injected into the ICP-MS instrument through a nebulizer, which converts the liquid sample into an aerosol. The ionized elements emit light at characteristic wavelengths, which are detected and quantified by a mass spectrometer. The concentration of trace elements in the samples were calculated by comparing the sample signals to the calibration curve.

### **Analysis of amino acids in culture media**.

The samples were centrifuged at 12000 rpm for 10 min and the supernatants were analyzed via a Waters Acquity^®^ UPLC H-Class-Xevo TQD system (MA, USA) equipped with electrospray ionization (ESI). HPLC was performed using an Acquity^®^ BEH C18 column (1.7 μm, 2.1 mm × 150 mm). The binary mobile phase consisted of solvent A (100% methanol) and solvent B (0.2% formic acid). The column gradient was as follows: 100% B/10 min, 66.6%B/0.5 min, returned to 100% B/1 min, and maintained at 100% B for another 0.5 min. The flow rate was 0.2 mL/min, the injection volume was 10 μL, and the column effluent was monitored by mass spectrometry. The mass spectrometric analysis was performed in multiple reactions monitoring (MRM) mode in positive ionization mode. The MS parameters were as follows: the dwell time was 0.02 s, and nitrogen was used as a desolvating gas at a flow rate of 600 L/h. The ionization source conditions were as follows: desolvation temperature 350°C; source temperature 150°C; collision gas (argon) flow 0.1 mL/min; and capillary voltage 3.0 kV. The parameters of mass analyzer were set as follows: the LM1 and HM1 resolutions were 15 and 15, respectively; as ion energy 1; the LM2 and HM2 resolutions were 15 and 15, respectively, and the ion energy was 2. The amino acids in the samples were identified on the basis of the mass-to-charge ratio.

The quantification of amino acids was conducted via a standard mix calibration curve, via the UPLC-MS/MS system controlled by Lynx software (Version 4.1, SCN 882). Data analysis was performed via the TargetLynx^™^ program. Each sample, which contained a mixure of amino acids at specific concentrations, was injected three times to ensure accuracy. The amino acid standard solution (AAS18, Sigma) included 2.5 μmol/mL of various amino acids such as L-alanine, L-arginine, and L-valine, with L-cystine at 1.25 μmol/mL. The samples were diluted in 0.1% formic acid in water for analysis. Using the MIDAS work flow, full scan linear ion trap MS/MS data confirmed the identity of the target analytes. MRM extracted ion chromatograms were used for all amino acids, especially at concentrations near their limits of detection (LODs), ensuring precise quantification and linearity. LODs were accurately calculated for all amino acids, and their concentrations were measured.

### Albumin ligand analysis and fractionation.

BSA was added to minimal growth requirements medium to a final concentration of 4.5 g/dL, left at RT for 0.5–1 hr and then filtered through Amicon 50 kDa MWCO ultracentrifugal filters (Merck) to remove albumin.

BSA after filtration (15 mL) was subjected to fractionation via C-18 solid-phase extraction (SPE, United States, Greece). After conditioning with 6 mL of MeOH and 6 mL of ultrapure water, 15 mL of the sample was applied, affording Fraction 1. Subsequently, with gradual elution (6 mL of 5% MeOH), Fraction 2 was obtained. Finally, Fractions 3 and 4 were obtained using 6 mL of 50% MeOH and 6 mL of pure MeOH, respectively. During the entire process, the flow rate was held constant at approximately 3 mL/min. The solvents and reagents used were of LC–MS grade (Merck, Greece).

For fraction component identification via GC–MS analysis, the obtained fractions (1–4) were derivatized with methyl-chloroformate (Sigma Aldrich, United States). Specifically, 300 μL of every fraction was mixed with 80 μL of pyridine, 200 μL of MeOH and 50 μL of methyl-chloroformate, vortexed for 30 sec and incubated for 6 min at room temperature. After incubation, 1 mL of hexane was added to each reaction mixture and extracted via the liquid–liquid extraction technique. Finally, the hexane phase was subjected to GC–MS analysis.

Fractions 1–4 were analysed via gas chromatography coupled with a single quadrupole mass spectrometry (GC–MS) analyser. GC–MS analysis was performed with an Agilent gas chromatography instrument (model 8860) system coupled to a mass spectrometer (model 5970) employing an electron ionization (EI) source.

The samples were qualitatively analysed, and caprylic acid (NIST 14.0 EI spectral library) was identified as the main component of fraction 2. Furthermore, the caprylic acid structure was verified via a reference standard (Sigma Aldrich, United States), and its concentration was quantitatively determined in all 4 fractions, following the same derivatization protocol and a standard solution calibration curve. The entire analysis was conducted in duplicate. The results are shown in Table x. Fraction 2 was the richest in caprylic acid, with an average concentration of 194.1 μg/mL, followed by fraction 3, with a much lower concentration of 15.2 μg/mL. In fractions 1 and 4, only traces of caprylic acid were detected.

Fraction 2 was also analysed via high-resolution mass spectrometry (HRMS) without any derivatization steps to verify the structure of caprylic acid. Specifically, an ultra-performance liquid chromatography (UPLC) system coupled with a high-resolution mass spectrometry (HRMS) analyser, especially a triple-TOF 5600+ (AB SCIEX) system, was employed in negative electrospray ionization (ESI) mode. LC analysis was performed on an ACQUITY H-Class UPLC system (Waters) equipped with a binary solvent manager and an FTN sample manager. A volume of 10 μL of fraction 2 was injected into the system and separated by a linear gradient containing water and ACN (mobile phases A and B, respectively, 5–100%), with the aqueous phase containing 0.1% formic acid. The solvents were of LC–MS grade and purchased from Merck (US). The analysis was performed with a Fortis Speedcore Biphenyl reversed-phase chromatography column (2.6 μm, 2.1 mm × 100 mm). The Triple-TOF platform was equipped with a DuoSpray ion source. The acquisition mode employed was data dependent, covering a mass range of 80–500 m/z. Specifically, the tripleTOF parameters for acquisition were as follows: source temperature of 450°C, source voltage of −4500 V, exhaust gas pressure of 50 psi, and curtain gas pressure of 35 psi. The caprylic acid structure was verified at m/z 143.1079 [M-H]- a, with a mass error of 1.1 ppm and rings and double bond equivalents of 1 **(Extended Data Figure 5).**

### Free fatty acid extraction from human plasma.

Free fatty acids were extracted from 100 mL of previously thawed plasma by the addition of 300 mL of cold methanol/ethanol (1:1) (Fischer Scientific, Pennsylvania, United States). C17-Sphinganine was added as an internal standard. The samples were vortexed for 1 min, incubated on ice for 5 min and centrifuged at 16,100 × g for 20 min at 4°C, after which 100 mL of the supernatant was transferred into liquid chromatography–mass spectrometry (LC–MS) vials for analysis.

### Free fatty acid extraction from the plasma of albumin-knockout mice.

Free fatty acids were extracted from 100 mL of previously thawed plasma by the addition of 300 mL of cold methanol (Fischer Scientific, Pennsylvania, United States). Next, 10 mL of 2 μM 9(S)-HODE-d4 solution (Cayman Chemical, Ann Arbor, MI, USA) was added as an internal standard. The samples were vortexed for 2 min and then centrifuged at 16,100 × g for 10 min at 4°C, after which 100 mL of the supernatant was transferred into LC–MS vials for analysis.

### Non-targeted analysis by LC–MS for free fatty acids.

LC-MS analysis was performed on a UHPLC system 1290 Infinity II (Agilent Technologies, Waldbronn, Germany) coupled with a 6546 QTOF MS detector in negative ESI mode. For separation, a volume of 1 mL was injected onto a Zorbax Rapid Resolution High Definition Extend-C18 column (Agilent Technologies, 2.1×50 mm, 1.8 μm) thermostated at 60°C. The flow rate was 0.6 mL/min, with a mobile phase composed of ultrapure Milli-Q water with 0.1% formic acid for A and acetonitrile with 0.1% formic acid for B. The chromatography gradient started from 5% B for the first min and increased to 80% B in 6.0 min, then to 100% by 11.0 min, and the starting conditions were returned to 1.0 min, allowing re-equilibration until 15.0 min. Data were collected in negative ESI ionization mode and operated in the range from m/z 100–600 and m/z 40–600 for MS/MS analysis via iterative Agilent mode. The nozzle voltage was set to 1000 V, and the capillary voltage was −4000 V with a scan rate of 1.2 scan/s. The drying gas was heated to 250°C at a rate of 12 L/min and a pressure of 52.0 psi. Additional heating was applied using sheath-heated gas up to 370°C with a flow rate of 11 L/min to improve ionization. For internal mass correction during data acquisition, one reference mass was infused continuously into the system throughout the whole analysis: m/z 112.9856 (proton-abstracted TFA anion). An external calibration with FA 18:0 was employed for semi-quantification of fatty acid species. Calibration curve samples were prepared at different concentrations (1.0, 2.5, 5.0, 10.0, 13.0, 17.0, and 20.0 ppm), and 9(S)-HODE-d4 was included as an internal standard at the same concentration as the serum samples. The raw data collected by LC–MS were reprocessed with MassHunter Profinder software version B.10.02. Calibration curves were built using normalized values of FA 18:0 by d4-9-HODE vs concentration in ppm. The results are expressed as the concentration in μM of the corresponding fatty acid in the serum.

### RNA isolation from *R. delemar* cells.

At the indicated time points of incubation in media with or without albumin (0 h, 3 h, and 6 h), the *R. delemar* cells were removed by scraping, centrifuged at 400 × *g*, and lysed with 450 μl of RLT buffer + β-mercaptoethanol via the RNeasy Plant Mini Kit (Qiagen). Each sample was subsequently sonicated using a sonication probe on ice for 20 × 1 s (set 40). Afterwards, RNA was isolated according to the manufacturer’s instructions.

### **RNA-seq data generation and analysis**.

RNA-seq libraries (strand-specific, paired end) were generated from total RNA by using a TruSeq RNA sample prep kit (Illumina). One hundred and fifty nucleotides of sequence were determined from both ends of each cDNA fragment using the HiSeq 4000 platform (Illumina). Sequencing reads were aligned to the reference genome (*R. delemar* 99–880) using HISAT^65^, and alignment les were used to generate read counts for each gene. Statistical analysis of differential gene expression was performed using the DEseq package from Bioconductor^66^. A gene was considered differentially expressed if the absolute log fold change was greater than or equal to 1 and the FDR value for differential expression was below 0.05. The RNA-seq analysis was performed in biological triplicate. RNA-sequence data was generated by Maryland Genomics at the Institute for Genome Sciences, University of Maryland School of Medicine.

### *In vivo* studies of fungal infection.

B6. Cg-Tg(FCGRT)32Dcr Alb^em12Mvw^ Fcgrt^tm1Dcr/MvwJ^, hereafter referred to as Alb^−/−^ (obtained from The Jackson Laboratory), and C57BL/6 mice were maintained in grouped cages in a high-efficiency air-filtered, environmentally controlled, virus-free facility (24°C, 12/12 h light/dark cycle) and fed a standard chow diet and water ad libitum. All experiments were approved by the local ethics committee of the University of Crete Medical School, Greece, in line with the corresponding National and European Union legislation (animal protocols 17/07/2017–147075 and 22/03/2023–90477). For virulence studies, 8–12-week-old, age- and sex-matched mice were infected via intratracheal instillation of ^1–5×106^ conidia of *R. oryzae* or *R. delemar*, and their survival was monitored for 14–21 days. After infection, two mice from each group were sacrificed for inoculum verification. For histopathological evaluation, lungs were collected at three different time points: 0 h, 6 h and 1 d post infection.

For the establishment of immunosuppression/neutropenia, 8–12-week-old, age- and sex-matched mice were administered 3 intraperitoneal injections of cyclophosphamide (Sigma–Aldrich, 150 mg/kg body weight on days −4 and −1, 100 mg/kg on day +3) and a subcutaneous injection of cortisone acetate (Sigma–Aldrich, 300 mg/kg on day −1), as previously described^31^. The mice were then infected intratracheally with 1×10^6^ spores of *R. delemar*. For the prophylactic model, 50 mg of FFA-free HSA was administered i.p. on days −6, −4 and −2 prior to pulmonary infection with *R. delemar*. For the therapeutic model, 25 mg of FFA-free HSA was administered i.p. daily for 6 consecutive days starting 6 h after pulmonary infection with *R. delemar*.

### Histopathology and immunohistochemistry/immunofluorescence studies.

The mice were euthanized, and their lungs were excised and fixed with 10% formalin before being embedded in paraffin and cut into 5–7 μm sections. Lung tissue sections were deparaffinized in xylene and rehydrated via an ethanol gradient (100%−70%). Hematoxylin and eosin or Grocott’s methenamine silver (GMS, Sigma–Aldrich) staining was performed for histopathology and fungal burden assessment, according to the manufacturers’ instructions.

For mucoricin immunostaining in the lung tissue, heat-induced antigen retrieval was performed to deparaffinize and rehydrate the lung sections through incubation in sodium citrate buffer (10 mM, 0.05% Tween 20, pH 6.0) for 40 min at 90–95°C via a steamer (Philips). The tissue sections were allowed to cool for 30 min, washed three times in PBS and permeabilized in 0.2% gelatin/Triton X-100 0.25% for 15 min at RT, followed by blocking in 5% BSA/5% normal goat serum (NGS)/PBS for 1 h at RT. The tissue slices were subsequently incubated at 4°C O/N with an anti-mucoricin antibody (2 mg/mL) in 1% BSA/1% NGS/PBS. Washing 3 times with PBS was followed by incubation with goat anti-rabbit CF488A or CF555 (1:500, Biotium) in the presence of 200 mg/mL CFW for 1 h at RT. The tissue sections were incubated for 10 min at RT with the nuclear dye TO-PRO-3 (1:2000, Thermo Fisher Scientific), washed thrice with PBS and quenched for auto fluorescence by incubation in a solution of 10 mM CuSO_4_/50 mM NH_4_Cl.

For mucoricin immunostaining in *R. delemar*, spores were cultured at 30°C in RPMI or RPMI supplemented with either 4.5 g/dL BSA or 1 mM caprylic acid for 6–8 hrs until they swelled. The swollen spores were fixed in 4% paraformaldehyde for 10 min, followed by permeabilization in 0.1% Triton X-100 in PBS for 10 min at RT. The permeabilized spores were blocked with 5% NGS in PBS for 1 h at RT and subsequently incubated with the anti-mucoricin antibody (1 mg/mL) for 2 h at RT. The spores were washed with Tris-buffered saline (0.01 M Tris HCl/0.15 M NaCl, pH 7.4) containing 0.05% Tween 20 and incubated with goat anti-rabbit CF488A (Biotium) diluted 1:500 in PBS for 1 h at room temperature.

For the analysis of FFA uptake by *R. delemar*, fungal spores were cultured in 5% ethanol/RPMI-MOPS supplemented with the indicated concentrations of oleic acid (Sigma–Aldrich) or oxidized oleic acid at 37°C for 5 h. The spores were harvested by scraping, collected in low-affinity 1.5 mL tubes, washed twice with PBS and fixed with methanol-free 4% formaldehyde at RT for 20 min. The spores were subsequently washed twice with PBS, stained with 0.5 μg/mL Nile red (Thermo Fisher Scientific) at RT for 20 min, washed again twice with PBS and stained with 200 μg/mL Calcofluor white at RT for 20 min. Flow cytometry data were acquired via a FACSCanto II system (BD Biosciences). All images were acquired via a spinning disk confocal system (Dragon y 200, Andor) equipped with a motorized inverted Nikon Eclipse Ti2-E microscope, an Andor Sona sCMOS 4.2B-6 camera, and four laser lines (405 nm, 488 nm, 561 mm and 633 nm). All images were obtained via z-stacks, deconvolved with Fusion software version 2.3.0.44 (Andor – Oxford Instruments) and further processed in Imaris 10.1 (Andor – Oxford Instruments) for contrast adjustment, area selection, color combining and scale bar addition. Certain images were acquired with a Leica TCS SP8 confocal microscope with a 63× lens and analysed with the use of Fiji/ImageJ.

Low-fluorescence immersion oil (Nikon) was used, and imaging was performed at RT. Quantitative analysis of fluorescence in confocal microscopy images was performed with IMARIS 10.0 (Oxford Instruments Andor).

### Western blot analysis.

The six elutions containing isolated human serum albumin were subjected to western blot analysis for assessment of the isolation protocol. Immunoblotting was performed according to the manufacturer’s instructions using simultaneous antibodies against human transferrin and albumin at a 1/1000 dilution, followed by incubation at 4°C O/N. The appropriate secondary antibodies were used at a 1/5000 dilution for 1 h at RT, and immunoblots were developed via chemiluminescence (ECL; Thermo Scientific).

### Antibodies.

Anti-mucoricin antibody was generated as previously described^10^. Antibodies against human albumin (# sc-365871) and transferrin (# sc-365871) were purchased from Santa Cruz (please provide cat). An antibody against mouse active caspase-3 was purchased from Cell Signaling (#9661).

### Statistical analysis.

All the statistical analyses were performed via Prism (GraphPad Software). A p value < 0.05 was considered statistically significant for all the variables tested. The data were processed and visualized via GraphPad Prism (version 9.04). The statistical methods used to determine significance and the p value of each graph are provided in the figure legends.

## Data Availability

All of the raw sequencing reads from this study are available at the NCBI Sequence Read Archive (SRA) under BioProject accession number PRJNA1141971. All the data that support the findings of this study are available from the corresponding author upon reasonable request.
